# Regulation of hepatic glucose production and AMPK by AICAR but not by metformin depends on drug uptake through the equilibrative nucleoside transporter 1 (ENT1)

**DOI:** 10.1111/dom.13455

**Published:** 2018-08-02

**Authors:** Lisa Logie, Zoe Lees, J. William Allwood, Gordon McDougall, Craig Beall, Graham Rena

**Affiliations:** ^1^ Division of Cellular Medicine Ninewells Hospital and Medical School, University of Dundee Dundee UK; ^2^ Environmental and Biochemical Sciences The James Hutton Institute Dundee UK; ^3^ Institute of Biomedical and Clinical Science University of Exeter Medical School Exeter UK

**Keywords:** 8CPT‐cAMP, AICAR, AMPK, ENT1, hepatic glucose production, hepatocyte, metformin

## Abstract

**Aim:**

Recently we have observed differences in the ability of metformin and AICAR to repress glucose production from hepatocytes using 8CPT‐cAMP. Previous results indicate that, in addition to activating protein kinase A, 8CPT‐modified cAMP analogues suppress the nitrobenzylthioinosine (NBMPR)‐sensitive equilibrative nucleoside transporter ENT1. We aimed to exploit 8CPT‐cAMP, 8CPT‐2‐Methyl‐O‐cAMP and NBMPR, which is highly selective for a high‐affinity binding‐site on ENT1, to investigate the role of ENT1 in the liver‐specific glucose‐lowering properties of AICAR and metformin.

**Methods:**

Primary mouse hepatocytes were incubated with AICAR and metformin in combination with cAMP analogues, glucagon, forskolin and NBMPR. Hepatocyte glucose production (HGP) and AMPK signalling were measured, and a uridine uptake assay with supporting LC‐MS was used to investigate nucleoside depletion from medium by cells.

**Results:**

AICAR and metformin increased AMPK pathway phosphorylation and decreased HGP induced by dibutyryl cAMP and glucagon. HGP was also induced by 8CPT‐cAMP, 8CPT‐2‐Methyl‐O‐cAMP and NBMPR; however, in each case this was resistant to suppression by AICAR but not by metformin. Cross‐validation of tracer and mass spectrometry studies indicates that 8CPT‐cAMP, 8CPT‐2‐Methyl‐O‐cAMP and NBMPR inhibited the effects of AICAR, at least in part, by impeding its uptake into hepatocytes.

**Conclusions:**

We report for the first time that suppression of ENT1 induces HGP. ENT1 inhibition also impedes uptake and the effects of AICAR, but not metformin, on HGP. Further investigation of nucleoside transport may illuminate a better understanding of how metformin and AICAR each regulate HGP.

## 1 INTRODUCTION

Stimuli that raise cyclic adenosine monophosphate (cAMP) levels in hepatocytes, including glucagon, induce de novo glucose production through gluconeogenesis and from glycogenolysis.^1^ Hyperglucagonaemia contributes to the chronic hyperglycaemia observed in type 1 (T1D) and type 2 (T2D) diabetes through poorly defined mechanisms. Raised intracellular cAMP activates downstream effectors including cAMP‐dependent protein kinase (PKA)^1^, ^2^ to control gluconeogenic flux through fructose‐1,6‐bisphosphatase. In addition, phosphorylation of cAMP‐response element binding protein (CREB) by PKA is believed to contribute to gluconeogenesis through increased expression of phosphoenolpyruvate carboxykinase (PEPCK) and glucose‐6‐phosphatase (G6‐Pase).

The hyperglycaemic effect of the glucagon/cAMP/PKA signalling pathway on liver cells has been studied using a number of different cAMP analogues, in combination with dexamethasone, to stimulate cAMP/PKA.^3^, ^4^, ^5^ The cAMP analogue dibutyryl cAMP (bucladesine, db‐cAMP), a cell‐permeable stabilized cAMP mimic that also inhibits phosphodiesterase (PDE) activity, is commonly used.^6^, ^7^ Treatment of cells with db‐cAMP causes a significant stimulation of PEPCK and G6‐Pase expression, which is inhibited by the addition of insulin in a dose‐dependent manner.^8^, ^9^, ^10^ 8‐(4‐chlorophenylthio)cAMP (8CPT‐cAMP), like db‐cAMP, is a membrane‐permeable cAMP analogue that stimulates PEPCK and G‐6‐Pase.^4^, ^11^ It tends to be more potent than cAMP, is more resistant to phosphodiesterase‐dependent hydrolysis and acts as a PDE inhibitor.^12^

Studies using cAMP analogues have shown previously that the T2D drug metformin and 5‐Aminoimidazole‐4‐carboxamide ribonucleoside (AICAR) repress cAMP‐stimulated hepatocyte glucose production (HGP).^13^, ^14^, ^15^ Inside cells, AICAR is phosphorylated by adenosine kinase to form ZMP, which then mimics AMP to activate AMPK.^16^ Metformin and AICAR are both activators of AMPK; however, earlier studies, including some carried out in mice where the catalytic subunits of AMPK are genetically ablated, demonstrated that suppression of HGP occurs independently of AMPK activation.^13^, ^14^, ^15^, ^17^, ^18^ Metformin is transported across hepatocyte cell membranes, at least in part, by an organic cation transporter (OCT) family of transporters.^18^, ^19^, ^20^ Previous studies using siRNA determined that AICAR is transported by ENT1 and CNT3 into human macrophages,^21^ whereas AICAR uptake into hepatocytes and its role concerning HGP is less clear. Initiating the current investigation, we observed that HGP was highly resistant to inhibition by AICAR only when 8CPT‐modified cAMP analogues were used to stimulate HGP. In contrast, repression of HGP by metformin was unaffected. 8CPT‐modified cAMP analogues have been shown previously to inhibit the equilibrative nucleoside transporter (ENT1), which is expressed in the liver^22^ and transports nucleosides across the plasma membrane, depending on the nucleoside concentration gradient.^23^ The inhibitory effect of 8CPT‐modified cAMP analogues on AICAR action prompted us to investigate the role of ENT1 in metformin and AICAR‐induced regulation of HGP using NBMPR, a highly selective ENT1 inhibitor.^24^ Our study indicates that suppression of ENT1 activity is sufficient to induce HGP. Moreover, the effects of AICAR, but not of metformin, on HGP are sensitive to ENT1 inhibition. These data highlight direct and indirect roles of ENT1 in modulating HGP.

## 2 MATERIALS AND METHODS

### 2.1 Materials

AICAR and 4‐Nitrobenzylthioinosine (NBMPR) were purchased from Tocris (Abingdon, UK). Dibutyryl cAMP, uridine and metformin were purchased from Sigma (Dorset, UK). LC‐MS materials were obtained from Fisher Scientific (Loughborough, UK) unless otherwise stated. Glucagon was obtained from Novo Nordisk (Bagsvaerd, Denmark). 8CPT‐cAMP was purchased from Calbiochem (Nottingham, UK) and 8CPT‐2‐Methyl‐O‐cAMP (8CPT‐2MeO‐cAMP) was purchased from Biolog Life Sciences Institute (Bremen, Germany). Donkey anti‐goat 680RD, donkey anti‐mouse 680RD, donkey anti‐rabbit 800CW secondary antibodies were purchased from LI‐COR Biosciences, UK. The GAGO glucose oxidase kit was purchased from Sigma. [5,6‐^3^H] uridine was purchased from Perkin Elmer (Beaconsfield, UK). pAMPK (Thr172) and total ACC antibodies were purchased from Cell Signalling Technologies (Leiden, UK). Total AMPK was purchased from Abcam (Cambridge, UK) and actin was purchased from Proteintech (Manchester, UK). pACC was made in‐house by Division of Signal Transduction Therapy (University of Dundee).

### 2.2 Isolation of mouse primary hepatocytes

Hepatocytes were isolated from adult female mice, essentially as described by Foretz et al.^14^ and in accordance with the Animals (Scientific Procedures) Act 1986. Following successful isolation, cell viability was determined by 0.04% Trypan blue staining and the cell number was determined using a haemocytometer. Cell viability of >90% was required before experimental use. Cells were plated at a density of 2.5 × 10^5^ cells per ml for all experiments.

### 2.3 Measurement of hepatocyte glucose production (HGP) from primary cells

HGP was measured, essentially as previously described,^25^ using mouse primary hepatocytes. Viable cells were allowed to settle for 4‐6 hours before serum starvation in M199 media containing dexamethasone (100 nM). Following overnight serum free conditions, cells were washed once in PBS before incubation for 8 hours in glucose‐free DMEM (Invitrogen, Paisley, UK) containing lactate (10 mM), pyruvate (1 mM), dexamethasone (100 nM) plus stimuli, as indicated in the Figure legends. Media was harvested for measurement of glucose and hepatocytes were lysed. The amount of glucose present in the media was measured using a GAGO glucose oxidase kit (Sigma). Glucose assays were performed in a 96‐well plate format. 50 μL of cell culture media was incubated with 100 μL of glucose oxidase/peroxidase assay reagent for 30 minutes at 37°C before the reaction was stopped by addition of 100 μL 12 N H_2_SO_4_. Absorbance was measured at 405 nm and the amount of glucose present was determined using a glucose standard curve generated in the same assay. The final glucose concentration was normalized to total protein content per well and data are presented as mg HGP per μg protein.

### 2.4 Cell lysis and western blotting

Cells were lysed in buffer containing Tris‐HCl (50 mM; pH 7.4), NaF (50 mM), sodium pyrophosphate (1 mM), EDTA (1 mM), EGTA (1 mM), NaCl (50 mM), sucrose (0.27 M), 1% (v/v) Triton‐X100, 0.1% (v/v) 2‐mercaptoethanol, sodium orthovanadate (1 mM), benzamidine (1 mM) and 0.1% (v/v) pefabloc, a serine protease inhibitor. Cells were allowed to lyse on ice for 30 minutes before scraping. Lysates were centrifuged at 13 K rpm for 15 minutes, at 4°C and were prepared in (final) 1x LDS buffer (Invitrogen) plus DTT (10 mM). Gels were run at 150 V for 90 minutes and were transferred onto nitrocellulose (GE Healthcare, Amersham, UK) for 3 hours at 75 V before blocking in 1% (w/v) BSA, TBS‐T for 30 minutes at room temperature. Membranes were incubated with primary antibodies and diluted 1:1000, with the exception of actin, which was diluted 1:5000, as indicated in the Figure legends, at 4**°**C overnight, with shaking before washing 3 × 10 minutes in 1xTBS‐T. Secondary antibodies (LI‐COR Biosciences, UK) were added at 1:5000 for 1 hour at room temperature, with shaking. Protein bands were visualized using the LI‐COR Odyssey infra‐red imaging system.

### 2.5 Uridine uptake assay

Primary mouse hepatocytes were incubated overnight in M199 media as described above. Cells were washed once in uridine uptake buffer (20 mM Tris‐HCl, 3 mM KH_2_PO_4_, 1 mM MgCl_2_.6H_2_O, 2 mM CaCl_2_, 5 mM Glucose, 130 mM NaCl, pH 7.4) before incubating at room temperature in 400 μL uridine uptake buffer containing DMSO (0.1% v/v), NBMPR (100 nM), 8CPT‐cAMP (100 μM), 8CPT‐2MeO‐cAMP (100 μM), db‐cAMP (100 μM) or glucagon (100 nM). After 15 minutes, 400 μL uridine uptake buffer containing compounds plus 0.1 μM (2 μCi/mL) ^3^H‐uridine was added. After 1 minute, uridine transport was stopped by washing cells five times in ice cold uridine uptake buffer containing cold uridine (1 mM). Cells were lysed in 200 μL 10% (w/v) SDS before scraping. The amount of uridine taken into the cell was determined by scintillation counting. Data are represented as percentage uptake compared with untreated control.

### 2.6 Liquid chromatography‐mass spectrometry (LC‐MS) analysis

Samples of culture media were transferred into 300 μL micro‐vials and capped (PN 60180‐507 and PN 60180‐516) (Thermo Scientific, Hemel Hempstead, UK). LC‐MS analysis was performed with a Thermo Accela 600 HPLC system, coupled with an Accela PDA detector (Thermo‐Fisher Ltd., Hemel Hempstead, UK). Samples were stored in the autosampler at 6°C and were analysed within 24 hours of preparation. The HPLC flow rate was 300 μL/min and the column (Gemini C6‐Phenol 110Ä, 150 × 2 mm, 5 μm particle size) (Phenomenex Ltd. Macclesfield, UK) was maintained at a temperature of 30°C. Solvent A was 5 mM ammonium acetate in deionized water (ELGA‐PureLab option‐Q) (Elga Ltd, High Wycombe, UK) adjusted to pH 3.5 with acetic acid and solvent B was HPLC grade acetonitrile (Fisher Scientific Ltd, Loughborough, UK). Prior to sample analysis, the column was conditioned with solvents A and B for 40 minutes. Samples (10 μL) were injected and eluted on a gradient programme of 100% A over 0‐2 minutes, 100%‐95% A over 2‐5 minutes; 95%‐55% A over 5‐25 minutes; 55% A‐100% B over 25‐26 minutes; hold 100% B over 26‐29 minutes, followed by 100% B‐100% A over 29‐30 minutes, followed by 100% A over 30‐35 minutes. HPLC needle washes were performed with 8:2 acetonitrile:water. An Accela PDA detector collected spectra from 200‐600 nm and monitored channels at 280, 365 and 520 nm.

PDA detector eluent was transferred to a Thermo LTQ‐Orbitrap XL mass spectrometry system operated under Xcalibur software (Thermo‐Fisher Ltd). For the first 3 minutes, the HPLC eluent flow was directed to waste, after which, over 3‐35 minutes, it was directed to the FT‐MS detector. Mass spectra were collected in full scan mode (*m/z* 80‐2000) at a mass resolution of 30 000 in both positive and negative ESI modes; however, positive mode provided the greatest sensitivity for AICAR. A second method was employed to validate compound identification through retention time and MS2 spectral matching to the reference standard. Here, data‐dependent MS2 CID fragmentation spectra (acquisition‐Q 0.25, activation time 25 ms, normalised collision energy 10, optimized for AICAR and metformin) were collected in the LTQ for the three most intense ions, as defined within the preliminary full FT‐MS scan. Both scan events generated “centroid” spectral data. Scan speeds of 0.1 seconds and 0.4 seconds were applied in the LTQ and FT‐MS, respectively. The automatic gain control was set to 1 × 10^5^ and 5 × 10^5^ for the LTQ and FT‐MS, respectively. ESI settings were optimized to allow efficient ionization and ion transmission with low in‐source fragmentation, spray voltage +4.5 kV, sheath gas 60, auxiliary gas 20, capillary voltage +10 V, tube lens voltage +80 V and capillary temperature at 280°C.

The MS detector was calibrated according to the manufacturer's instructions, after which it was tuned to optimize detection of ions in the mid *m/z* 80‐2000 range. All samples were run in a randomized order, with control and blank samples interspersed at every six analyses and at the beginning and end of the run, to monitor HPLC‐MS background and target compound carryover.

A stock solution of 750 μM AICAR (99% + pure) (Sigma Chemical Co. Ltd) was prepared in fresh culture media (Dulbecco's Modified Eagle Medium 1X) (Gibco Life Technologies Corp., Paisley, UK), after which it was serially diluted in culture media to provide calibration standard curves. Extracted ion chromatograms for AICAR were generated within Xcalibur Qual Browser software; peak areas were extracted applying the GENESIS peak detection algorithm and raw extracted peak areas were transferred to Microsoft Excel.

### 2.7 Statistics

All statistical analyses were performed using GraphPad Prism version 6.0. Mean glucose values were taken from each experiment, and in one experiment were normalized to control, which was set at 100% to account for variability among different hepatocyte preparations. HGP and western blotting data were analysed by ANOVA. For the uridine uptake assay, data were expressed as percentage of control uptake and were analysed by one sample t‐test, with control set to 100%.

## 3 RESULTS

### 3.1 8CPT‐cAMP inhibits AICAR, but not metformin, repression of HGP

**Figure 1:**
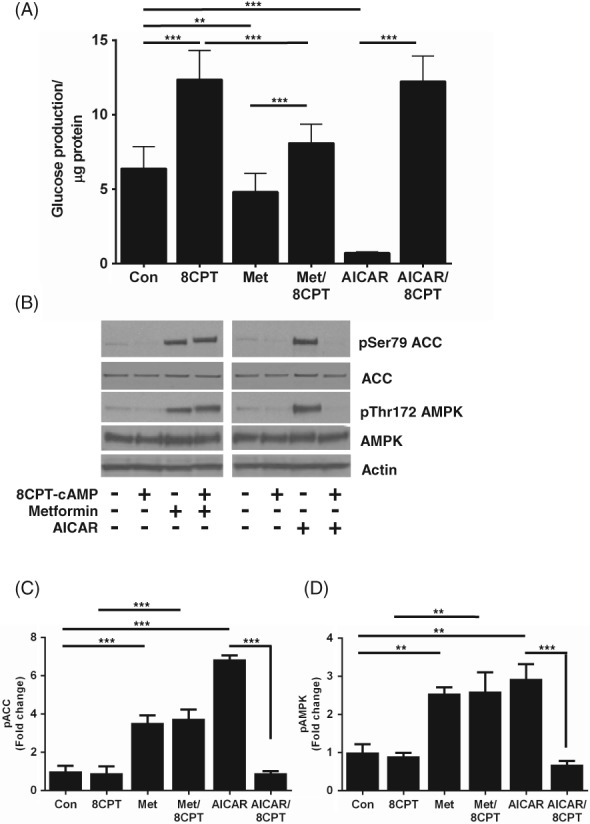
8CPT‐cAMP inhibits the ability of AICAR, but not of metformin, to suppress HGP in primary hepatocytes. A, HGP was measured from primary mouse hepatocytes after 8 hours of incubation, with or without 8CPT‐cAMP (100 μM), in the presence or absence of metformin (Met) (250 μM) or AICAR (250 μM). B, Representative immunoblots of pThr172 AMPK and pSer79 ACC from primary hepatocytes treated with metformin (250 μM), AICAR (250 μM) ± 8CPT‐cAMP (100 μM). C, D Densitometric analysis of phosphorylation of ACC (C) and AMPK (D), expressed as fold change of control (n = 3). All data are expressed as mean ± SEM and were analysed by ANOVA (*P < 0.05; **P < 0.01; ***P < 0.001). Abbreviation: Con, control

Primary mouse hepatocytes were serum starved overnight before incubating with 8CPT‐cAMP (100 μM), in the presence or absence of AICAR (250 μM) or metformin (250 μM), for 8 hours. Consistent with previous work,^26^ incubation of hepatocytes with 8CPT‐cAMP increased HGP. AICAR (250 μM) caused an almost complete inhibition of basal, but not 8CPT‐cAMP‐stimulated HGP, suggesting that 8CPT‐cAMP interferes with the ability of AICAR to inhibit HGP. In contrast, although metformin (250 μM) caused only a modest repression of basal HGP, it significantly repressed 8CPT‐cAMP‐stimulated HGP (Figure 1A). Metformin and AICAR both activate AMPK. Previous work, including experiments in which AMPK activity was genetically ablated, indicate that AMPK is not essential for regulation of HGP, although both drugs activate AMPK.^13^, ^14^, ^15^, ^17^, ^18^ In the current study, we used AMPK activation purely as a marker of drug entry into the cell. 8CPT‐cAMP prevented increases in phosphorylation of acetyl CoA carboxylase (ACC) and AMPK by AICAR, but not by metformin (Figure 1B,D), indicating that activation of the AMPK signalling pathway by AICAR was selectively blocked by 8CPT‐cAMP.

### 3.2 Inhibition of AICAR's effects depends on the 8CPT moiety of 8CPT‐cAMP

**Figure 2:**
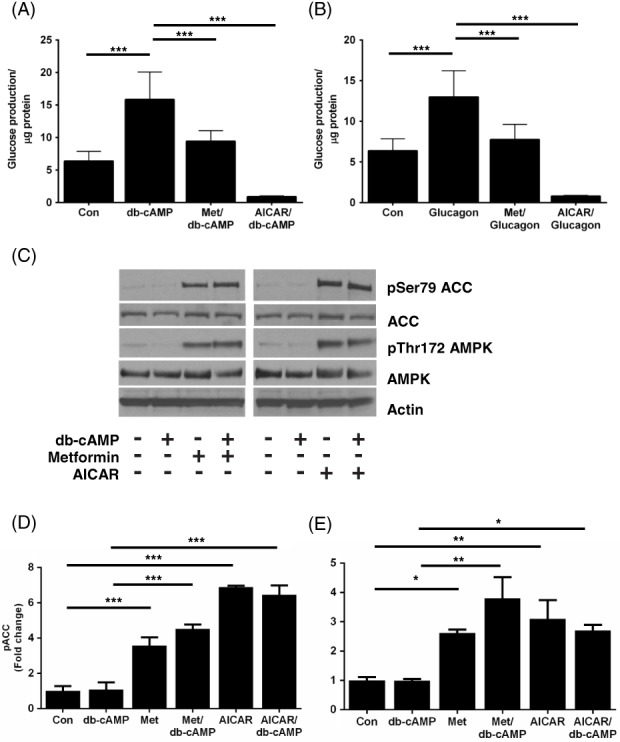
Dibutyryl cAMP and glucagon do not inhibit the effect of AICAR on HGP or AMPK signalling. Primary mouse hepatocytes were incubated for 8 hours with either dibutyryl cAMP (db‐cAMP, 100 μM) A, or glucagon (100 nM) B, in the presence or absence of metformin (Met) (250 μM) or AICAR (250 μM). The amount of glucose produced was measured as in Figure 1A. C, Immunoblots showing pT172 AMPK and pS79 ACC phosphorylation in response to db‐cAMP (100 μM). D, E, Densitometric analysis of phosphorylation of ACC (D) and AMPK (E), expressed as fold change of control. Data are expressed as mean ± SEM from three separate experiments and were analysed by ANOVA (*P < 0.05; **P < 0.01; ***P < 0.001 in post hoc comparisons). Abbreviation: Con, control

**Figure 3:**
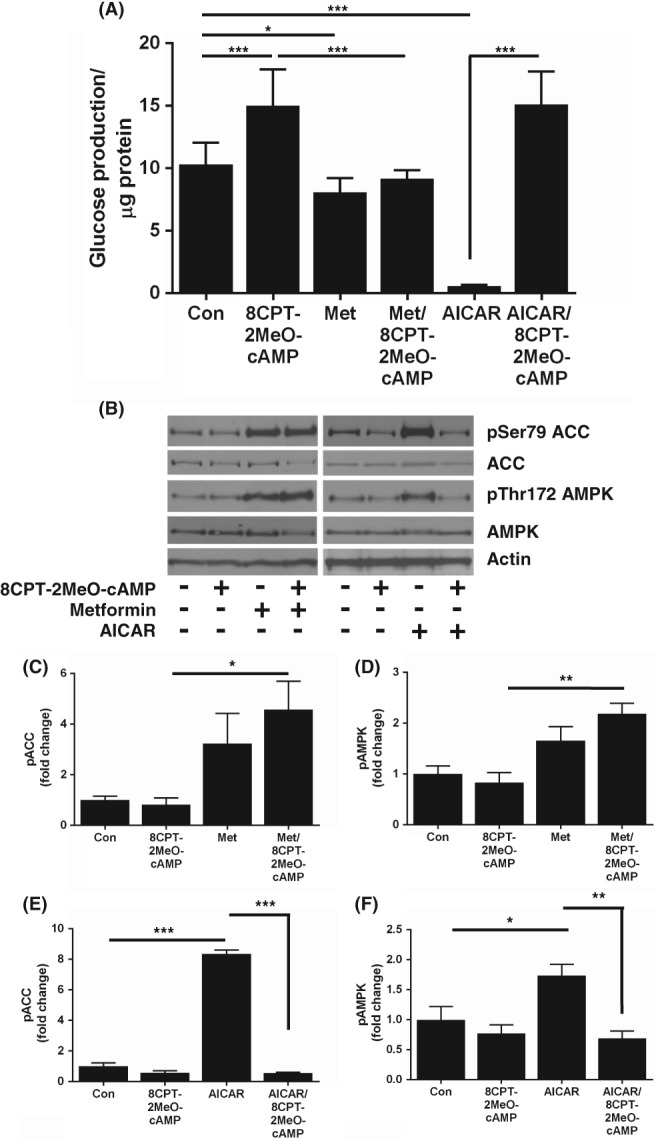
Suppression of HGP and AMPK signalling by AICAR is inhibited by the 8CPT‐modified cAMP analogue 8CPT‐2‐methyl‐O‐cAMP. Primary hepatocytes were treated for 8 hours with metformin (Met) (250 μM) or AICAR (250 μM) ± 8CPT‐2MeO‐cAMP (100 μM) A, Media were harvested and HGP was measured as in Figure 1A (*n* = 4). B, Representative immunoblots of pThr172 AMPK and pSer79 ACC phosphorylation in lysates treated with metformin (Met) (250 μM) or AICAR (250 μM) ± 8CPT‐2MeO‐cAMP (100 μM). C‐F, Densitometric analysis of ACC (C, E) and AMPK (D, F) phosphorylation, expressed as fold change of control (*n* = 4). Data are expressed as mean ± SEM and were analysed by ANOVA (*P < 0.05; **P < 0.01; ***P < 0.001 in pairwise comparisons). Abbreviation: Con, control

We investigated the ability of AICAR to suppress HGP in the presence of db‐cAMP and glucagon to induce endogenous cAMP. In contrast to 8CPT‐cAMP, AICAR significantly repressed HGP in the presence of db‐cAMP (100 μM) (Figure 2A) and glucagon (100 nM) (Figure 2B). In addition, the effects of AICAR on AMPK and ACC phosphorylation were not modified by db‐cAMP and glucagon (Figure 2C,E). AICAR also suppressed HGP induced by the adenylate cyclase activator forskolin (100 μM) (Figure S1). To determine whether other 8CPT‐modified cAMP analogues inhibit the effect of AICAR on HGP and AMPK signalling, we utilized 8CPT‐2MeO‐cAMP. We incubated primary mouse hepatocytes with 8CPT‐2MeO‐cAMP and found that, similar to 8‐CPT‐cAMP, 8CPT‐2MeO‐cAMP blocked repression of HGP by AICAR (Figure 3A). When the effects of 8CPT‐2MeO‐cAMP on AMPK and ACC phosphorylation were studied, we found that, like 8CPT‐cAMP, 8CPT‐2MeO‐cAMP selectively inhibited AICAR's, but not metformin's ability to stimulate AMPK signalling (Figure 3B,F).

### 3.3 AICAR uptake into hepatocytes is mediated by equilibrative nucleoside transporter 1 (ENT1)

**Figure 4:**
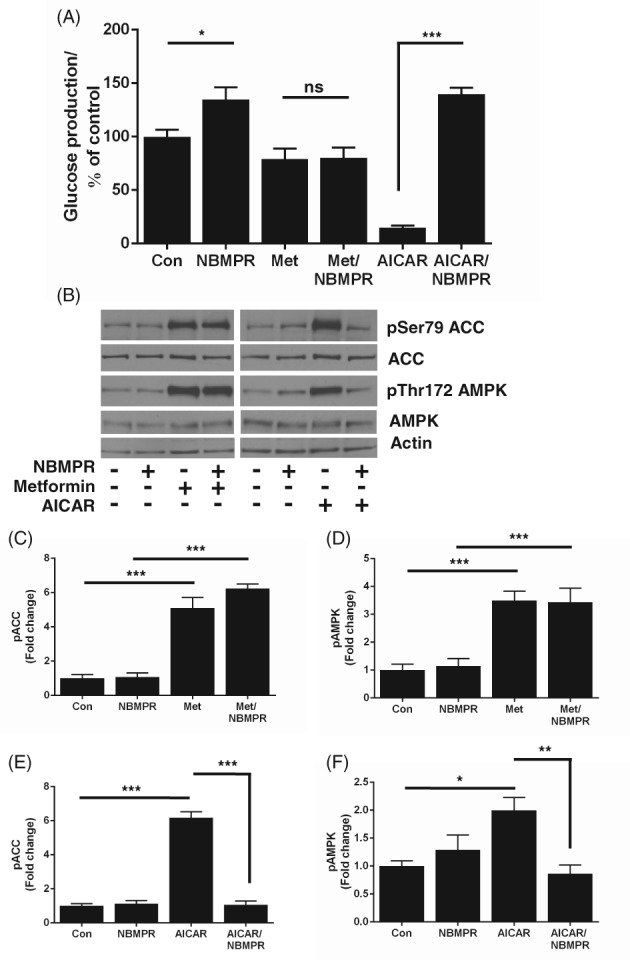
AICAR uptake into hepatocytes is mediated by equilibrative nucleoside transporter 1 (ENT1). A, HGP was measured after incubation for 8 hours with the selective ENT1 inhibitor NBMPR (100 nM) ± metformin (250 μM) or AICAR (250 μM). The amount of glucose produced was normalized to protein content and expressed as % control. B, Representative immunoblots measuring pThr172 AMPK and pSer79 ACC by metformin (250 μM) or AICAR (250 μM) ± NBMPR (100 nM). C‐F, Densitometric analysis of ACC (C, E) and AMPK (D, F) phosphorylation, expressed as fold change of control. Data are taken from 4 separate experiments and shown as mean ± SEM, (*P < 0.05; **P < 0.01; ***P < 0.001 in pairwise comparisons). Abbreviations: Con, control; ns, not significant

**Figure 5:**
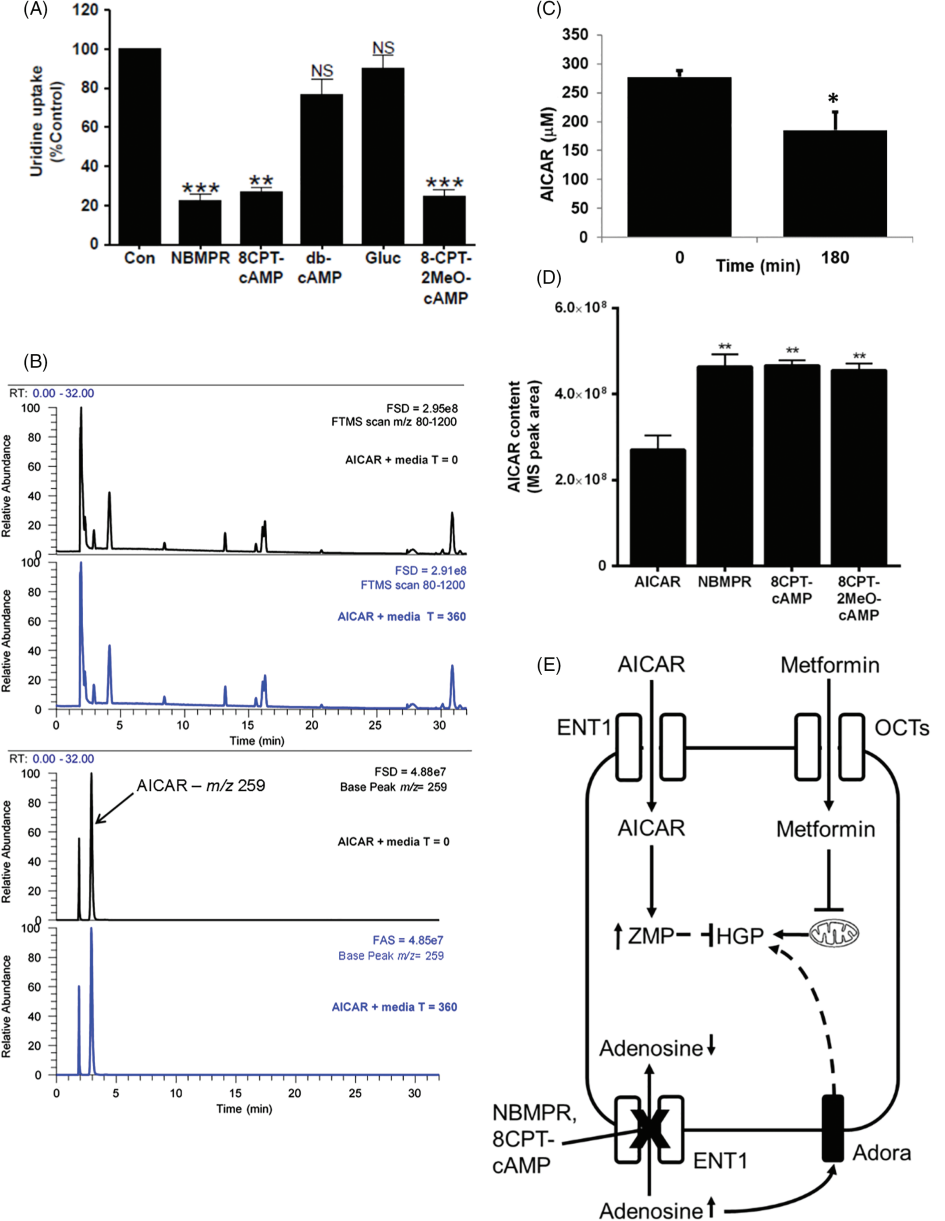
Nucleoside transport via equilibrative nucleoside transporter 1 (ENT1) is inhibited by 8CPT‐modified cAMP analogues. Uridine uptake into hepatocytes was measured using ^3^H‐uridine uptake assay. Hepatocytes were incubated for 15 minutes with NBMPR (100 nM), 8CPT‐cAMP (100 μM), dibutyryl cAMP (100 μM), glucagon (100 nM) or 8CPT‐2MeO‐cAMP (100 μM) before measuring ^3^H transport into the cell for 1 minute. Data are expressed as mean ± SEM and are represented as % uridine uptake relative to control (*n* = 3) **P < 0.01, ***P < 0.001). B, LC‐MS analysis of AICAR in medium without cells at 0 and 6 hours of incubation. The top two traces show the similarity between total MS signal at 0 and 6 hours of incubation. The bottom two traces show the signal at the selected *m/z* 259 for AICAR and demonstrate that AICAR is stable under these conditions. The full‐scale deflection of the MS detector is given in the top right corner of each trace. C, AICAR disappearance from medium in the presence of primary hepatocytes. AICAR in media was measured by LC‐MS and expressed as μM AICAR. Data are expressed as mean ± SEM and were analysed by ANOVA (*P < 0.05 in pairwise comparisons). D, Effect of NBMPR, 8CPT‐cAMP and 2MeO‐8CPT‐cAMP on AICAR disappearance from the medium. AICAR in media was measured by LC‐MS and expressed as peak areas in MS detector units. Data are expressed as mean ± SEM and analysed by ANOVA (*P < 0.05 and **P < 0.01 in pairwise comparisons). E, Schematic representation of AICAR and metformin entry into hepatocytes and consequences of equilibrative nucleoside transporter (ENT1) inhibition. Upper section: AICAR enters the hepatocyte through ENT1, where it is converted to AICAR monophosphate (ZMP), resulting in suppression of HGP through AMPK‐independent targets; metformin enters hepatocytes, at least in part, through organic cationic transporters (OCT) and inhibits HGP through mechanisms including mitochondrial inhibition, affecting adenine nucleotide levels/ratios. Lower section: NBMPR inhibits adenosine uptake into hepatocytes through ENT1, raising HGP which is possibly mediated, at least in part, by an adenosine receptor (Adora)‐mediated process. Abbreviations: Con, control; NS, not significant

Previous studies have shown that 8‐CPT‐modified cAMP analogues are potent inhibitors of the equilibrative nucleoside transporter 1 (ENT1),^23^ which, in brain slices, was previously found to mediate AICAR uptake.^27^ This led us to test whether AICAR uptake into hepatocytes may be blocked by inhibition of ENT1. We incubated primary mouse hepatocytes with AICAR and metformin in the presence of the selective ENT1 inhibitor NBMPR (Figure 4A). This compound was sufficient to promote HGP alone, but completely blocked the action of AICAR on HGP (Figure 4B). The effect of metformin on AMPK and ACC phosphorylation was not modified by the presence of NBMPR (Figure 4B,D), whereas AICAR failed to alter AMPK or ACC phosphorylation in the presence of NBMPR (Figure 4B,E,F). These data strongly suggest that AICAR uptake into mouse hepatocytes is mediated by ENT1. To confirm that 8CPT‐cAMP and 8CPT‐2MeO‐cAMP inhibit ENT1 in hepatocytes, we performed an uptake assay, using radiolabelled uridine as a tracer and a known ENT1 substrate to measure ENT1 activity in the absence and presence of NBMPR, 8CPT‐cAMP, 8CPT‐2MeO‐cAMP, db‐cAMP and glucagon. We found that ^3^H‐uridine uptake was inhibited by NBMPR, 8CPT‐cAMP and 8CPT‐2MeO‐cAMP but was not inhibited by db‐cAMP or glucagon (Figure 5). To reinforce these findings, we carried out LC‐MS analysis of AICAR depletion from cellular medium. A scan of cell culture medium without AICAR is presented online (Figure S2A). On the same column, AICAR ran as a single peak at 2.91 minutes (Figure S2B‐S2D). We then established that addition of AICAR alone to the medium was stable up to 24 hours, with LC‐MS traces superimposable (0 vs 360 minutes) (Figure 5B), indicating that AICAR is not degraded over this time‐period. In contrast, in media harvested from cells treated with AICAR, we observed AICAR loss from the medium (Figure 5C), which was blocked by NBMPR, 8CPT‐cAMP and 2MeO‐8CPT‐cAMP (Figure 5D).

## 4 DISCUSSION

Regulation of HGP is key to maintaining adequate blood glucose control in T1 and T2 diabetes. Glucagon and a number of cAMP analogues, including 8CPT‐cAMP and db‐cAMP, are used in pre‐clinical studies to mimic HGP in the fasted state, allowing the liver‐specific effects of metformin and AICAR on HGP to be studied in vitro. This study was prompted by our observation that 8CPT‐cAMP selectively blocks suppression of HGP and activation of AMPK signalling in response to AICAR, but not in response to metformin. Db‐cAMP and glucagon stimulated HGP, which was reversed by AICAR. A second 8CPT‐modified analogue, 8CPT‐2MeO‐cAMP produced a similar selective block of the effect of AICAR on both HGP and AMPK signalling. Earlier investigations found that 8CPT‐cAMP and 8CPT‐adenosine did not inhibit the binding of [^3^H]NBMPR to PC12 cells^23^ and consequently, the mechanism of 8CPT‐cAMP/8CPT‐adenosine dependent inhibition of ENT1 is uncertain but is probably related to the fact that 8CPT‐cAMP is more lipophilic than cAMP by one order of magnitude.^28^ This significant off‐target effect of 8CPT‐modified cAMP analogues suggests that caution should be exercised in future studies using 8CPT‐cAMP to stimulate HGP. In the current study, however, we exploited this action of 8CPT‐modified cAMP analogues to compare hepatic actions of AICAR and metformin.

We excluded a role for PKA/Epac activation in the blocking effect of 8CPT‐modified cAMP analogues on AICAR‐dependent suppression of HGP, on the assumption that cAMP does not affect the inhibitory effect of AICAR on HGP, when raised by the physiological inducer of cAMP, glucagon, and by the adenylate cyclase activator, forskolin. Importantly, it has been reported previously that 8CPT‐modified cAMP analogues inhibit nucleoside transporters,^23^ which are divided into two different families: constitutive nucleoside transporters (CNT) (SLC28), of which there are three members (CNT1‐3) or equilibrative nucleoside transporters (ENT) (SLC29), of which there are four members (ENT1‐4).^29^ These nucleoside transporters are responsible for transporting nucleosides such as adenosine and uridine into the cell. In the brain, AICAR elevates adenosine levels, thought to be mediated by competition for or blockade of ENT1.^27^ Therefore, we used a highly selective inhibitor of ENT1, NBMPR, to demonstrate that ENT1 inhibition is sufficient to block the effect of AICAR on HGP and AMPK signalling. Molecular docking data are unavailable for 8CPT‐cAMP analogues docking to ENT1 but in contrast, robust 3D‐Quantitative Structure‐Activity Relationship analysis is available for NBMPR/ENT1 interaction.^30^, ^31^, ^32^ NBMPR did not alter the effects of metformin on AMPK and HGP. At the nanomolar concentrations used in the current study, NBMPR is highly specific for ENT1 which has a unique high‐affinity binding site for this drug.^24^ In recombinant systems, NBMPR inhibits ENT1 7000‐fold more potently than ENT2,^24^, ^33^ and it does not inhibit CNT3.^34^ In rat hepatic membrane preparations, NBMPR is without effect on Na^+^‐dependent (concentrative) adenosine transport activity.^35^ To further validate that AICAR was taken up via ENT1, we used ^3^H‐uridine as a tracer. We confirmed that 8CPT‐cAMP analogues and NBMPR inhibit uridine uptake into hepatocytes. We then cross‐validated with LC‐MS, demonstrating that AICAR is depleted from media harvested from AICAR‐treated hepatocytes, which was prevented by NBMPR and 8CPT‐modified cAMP analogues. Using an additional control, we did not observe any major degradation products of AICAR in the medium, indicating that AICAR depletion is most likely the result of cellular uptake. The difference between AICAR and metformin sensitivity to NBMPR is consistent with metformin being taken into the cell via OCT transporters, as reported previously.^19^, ^20^ Our findings may explain previous observations that required the use of very high (2 mM) concentrations of AICAR to inhibit 8CPT‐cAMP‐induced HGP.^11^ Taken together, our data suggest that 8CPT‐modified cAMP analogues and NBMPR inhibit ENT1 to block AICAR entry, preventing AICAR‐mediated inhibition of HGP and activation of AMPK (Figure 5E).

Direct ENT1 inhibition with NBMPR which, in other tissues, raises extracellular adenosine levels,^36^, ^37^ was sufficient to increase HGP (Figure 5E). This is probably mediated indirectly by activation of adenosine receptor‐mediated glycogenolysis or gluconeogenesis.^38^ In addition to adenosine, ENT1 also transports uridine, which promotes feeding via activation of hypothalamic uridine diphosphate receptor, P2Y6,^39^ and which may also contribute AMPK‐independent effects of AICAR on metabolism. Several clinically used drugs inhibit ENT1 as a secondary mode of action, including rosuvastatin^40^ and dipyridamole.^41^ Interestingly, rosuvastatin increases HbA1c in individuals with and without diabetes,^42^ and dipyridamole increases glycaemia in mice.^43^ Taken together, these data suggest that therapeutic alterations in purine salvage may produce a clinically significant alteration in HGP via modulation of adenosine receptors.

In summary, we report for the first time that suppression of ENT1 induces HGP. ENT1 inhibition also impedes uptake and the effects of AICAR, but not of metformin, on HGP. These data delineate the differing pathways by which AICAR and metformin regulate HGP, and more study is required to determine whether exploitation of these pathways can be used for further therapeutic intervention in diabetes.

## Supporting information


**Fig. S1** Forskolin does not inhibit the effect of AICAR on glucose production or AMPK signalling. Primary mouse hepatocytes were incubated for 8 hours with forskolin (FSK; 100μM) in the presence or absence of metformin (Met; 250 μM) or AICAR (250 μM). The amount of glucose produced was measured as in Figure 1A. Data is expressed as mean ± SEM from three separate experiments and analysed by ANOVA (*P < 0.05, **P < 0.01, ***P < 0.001 in pairwise differences).
**Fig. S2** Supporting mass spectrometry data. A, Base peak MS trace showing elution of *m/z* 259 peak at 2.94 minutes, B, MS spectra of m/z 259, and C, MS^2^ spectra of m/z 259 showing major fragments. The exact mass *m/z* [M + H] = 259.1037 gave a predicted formula C_9_H_15_N_4_O_5_ at ppm < 1 and this matched the actual formula, C_9_H_14_N_4_O_5_. The major MS^2^ fragments at 127 and 110 reflect neutral loss of 132 (ribose) and 149. These agree with previous work (Thomas et al., 2010) and match those reported in the PubChem record (266934) for this compound. Levels of AICAR in triplicate samples were quantified by reference to a standard curve of peaks areas obtained using the resident Xcalibur software from 0‐500 μM of AICAR in the cell media. The stability of the AICAR was tested in cell media without cells but under the same conditions as used in the cell studies noted previously.
