# Supplementary material for: Regulation of hepatic glucose production and AMPK by AICAR but not by metformin depends on drug uptake through the equilibrative nucleoside transporter 1 (ENT1)
Source: Diabetes Obes Metab. 2018 Aug 2;20(12):2748–58. doi: 10.1111/dom.13455 (PMC6282725; doi:10.1111/dom.13455)
Supplement: Supplementary file 1 — Fig. S1 Forskolin does not inhibit the effect of AICAR on glucose production or AMPK signalling. Primary mouse hepatocytes were incubated for 8 hours with forskolin (FSK; 100μM) in the presence or absence of metformin (Met; 250 μM) or AICAR (250 μM). The amount of glucose produced was measured as in Figure 1A. Data is expressed as mean ± SEM from three separate experiments and analysed by ANOVA (*P < 0.05, **P < 0.01, ***P < 0.001 in pairwise differences). Fig. S2 Supporting mass spectrometry data. A, Base peak MS trace showing elution of m/z 259 peak at 2.94 minutes, B, MS spectra of m/z 259, and C, MS2 spectra of m/z 259 showing major fragments. The exact mass m/z [M + H] = 259.1037 gave a predicted formula C9H15N4O5 at ppm < 1 and this matched the actual formula, C9H14N4O5. The major MS2 fragments at 127 and 110 reflect neutral loss of 132 (ribose) and 149. These agree with previous work (Thomas et al., 2010) and match those reported in the PubChem record (266934) for this compound. Levels of AICAR in triplicate samples were quantified by reference to a standard curve of peaks areas obtained using the resident Xcalibur software from 0‐500 μM of AICAR in the cell media. The stability of the AICAR was tested in cell media without cells but under the same conditions as used in the cell studies noted previously. [file DOM-20-2748-s001.docx]

**Supplementary data**

**Fig. S1. Forskolin does not inhibit the effect of AICAR on glucose production or AMPK signalling.** Primary mouse hepatocytes were incubated for 8 hours with forskolin (FSK; 100μM) in the presence or absence of metformin (Met; 250 μM) or AICAR (250 μM). The amount of glucose produced was measured as in Fig. 1A. Data is expressed as mean +/- SEM from three separate experiments and analysed by ANOVA (*p<0.05, ** p<0.01, *** p<0.001 in pairwise differences).

**Fig. S2. Supporting mass spectrometry data.**

(A) Base peak MS trace showing elution of *m/z* 259 peak at 2.94 min,

(B) MS spectra of m/z 259, and (C) MS^2^ spectra of m/z 259 showing major fragments. The exact mass *m/z* [M+H] = 259.1037 gave a predicted formula C_9_H_15_N_4_O_5_ at ppm < 1 and this matched the actual formula, C_9_H_14_N_4_O_5_. The major MS^2^ fragments at 127 and 110 reflect neutral loss of 132 (ribose) and 149. These agree with previous work (Thomas et al., 2010) and match those reported in the PubChem record (266934) for this compound. Levels of AICAR in triplicate samples were quantified by reference to a standard curve of peaks areas obtained using the resident Xcalibur software from 0 – 500 μM of AICAR in the cell media. The stability of the AICAR was tested in cell media without cells but under the same conditions as used in the cell studies noted previously.

**Reference**

A.Thomas, S. Beuck, J. Christian Eickhoff, S. Guddat, O. Krug, M. Kamber, W Schänzer & M. Thevis (2010) Quantification of urinary AICAR concentrations as a matter of doping controls. Anal Bioanal Chem (2010) 396:2899–2908


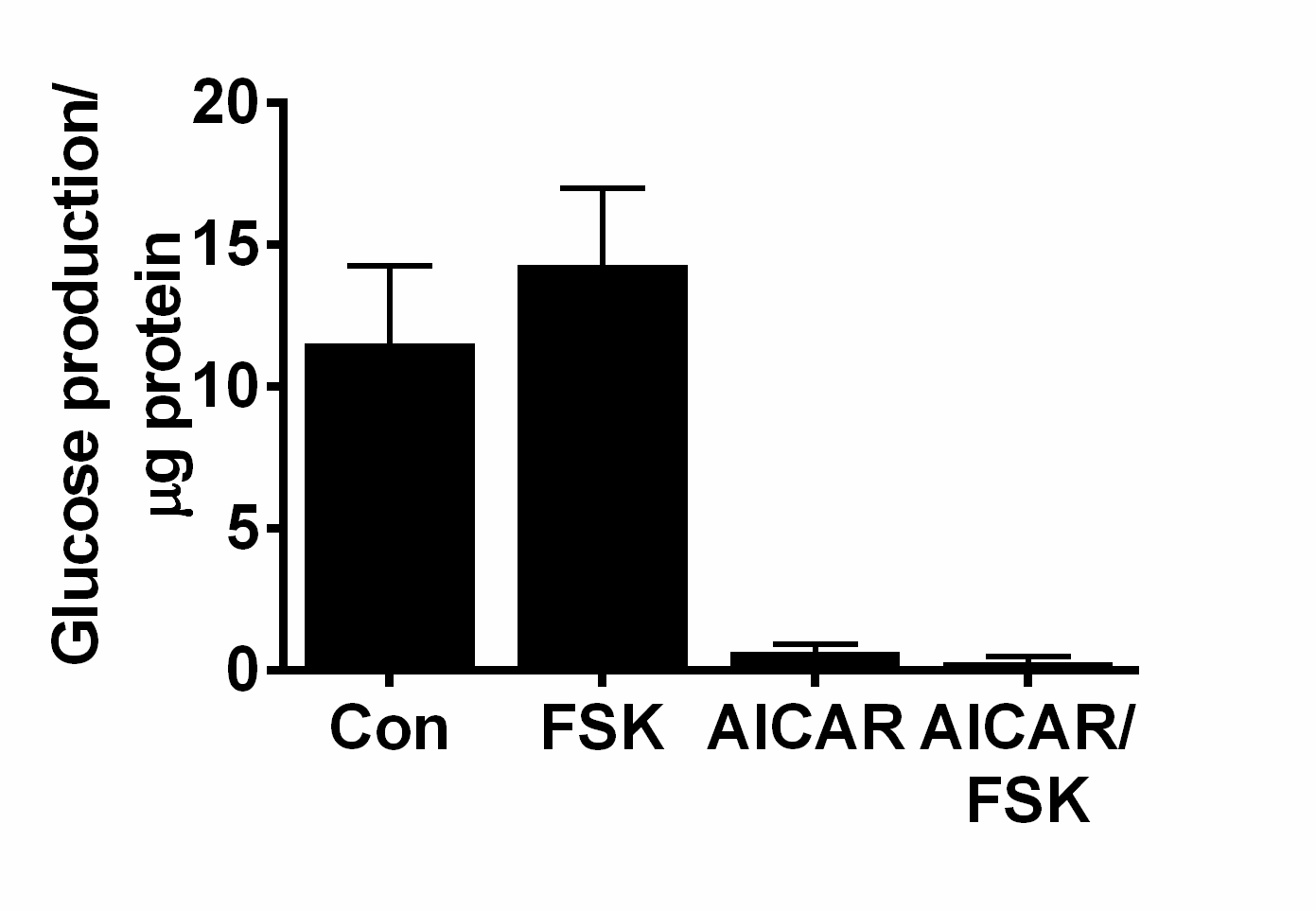


**Supplementary Fig. 1**

*******

*******

*******

**Supplementary Fig. 2**

**A**

5

10

15

20

25

Time (min)

0

10

20

30

40

50

60

70

80

90

100

Relative Abundance

2.94

FSD = 2.12e7

Base Peak *m/z* = 259

**AICAR**

FSD = 2.12e7

FTMS + c ESI Full ms [80-2000]

100

150

200

250

300

350

400

450

500

550

600

*m/z*

0

10

20

30

40

50

60

70

80

90

100

Relative Abundance

**259.10**

**517.20**

317.16

449.34

297.06

195.09

127.06


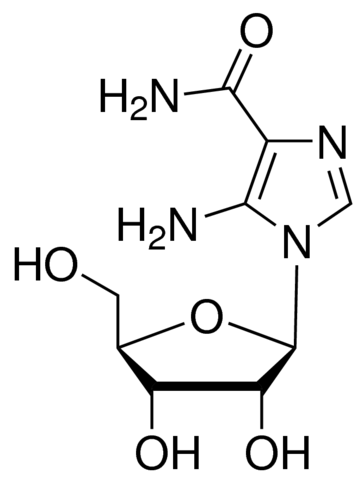


**B**

AICAR MS^2^

FSD = 4.56e5:

FTMS + c ESI d Full ms^2^ 259.10@cid30.00 [80-270]

80

100

120

140

160

180

200

220

240

260

*m/z*

0

20

40

60

80

100

Relative Abundance

**127.02**

128.01

**110.02**

241.06

223.09

169.01

205.05

97.02

**C**
